# A rare image phytobezoar in stomach

**DOI:** 10.11604/pamj.2015.20.32.5844

**Published:** 2015-01-13

**Authors:** Kenan Ahmet Turkdogan, Mehmet Yigit

**Affiliations:** 1 Bezmialem Vakif University, Department of Emergency Medicine, Istanbul, Turkey

**Keywords:** Conservative treatment, emergency medicine, phytobezoar

## Image in medicine

42-year-old female patient came with complaints of 5 days abdominal pain, nausea, vomiting and constipation to our emergency service. No property was determined when chronic diseases of our patient, drugs used and history of surgery was questioned. It was learned that the purpose of slimming diet rich in fiber has been made for 10 days. Increase in bowel sounds on auscultation in the upper quadrant of the abdomen, while there was a decrease in the lower quadrant. The patient's vital signs were normal with normal laboratory values. Nasogastric tube was inserted in patients with vomiting, abdominal distension and constipation. There were air and liquid levels in the intestine in her direct abdominal radiographs, there was severely distended stomach and the gastric lumen had been filling with opacities (A). Intense soft tissue and isodense density which fills the lumen are available in the abdominal tomography, fluid and air leveling were observed in intestinal (B). Oral in take was stopped and intravenous hydration were started. The patient was discharged on the 5th day of hospitalization with a poor diet in fiber. In patients without under lying chronic disease and a history of previous surgery, the patient can be given the chance of treatment with conservative method.

**Figure 1:**
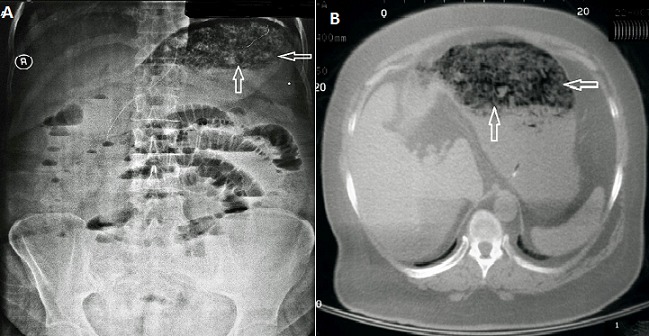
A) in the patient's direct abdominal radiographs, there was severely distended stomach and the gastric lumen had been filling with opacities; B) intenses of tissue and isodense density which fills the lumen are available in the abdominal tomography

